# A case of thyroxine (T4) toxicosis complicated by thyroid storm with an unusual precipitant 

**DOI:** 10.22088/cjim.11.2.231

**Published:** 2020

**Authors:** Akolade O. Idowu, Oluwaseyitan Andrew Adesegun, Bamikole Osibowale, Theophilus Ajiro, Daniel Ezuduemoih, Ayokunle Osonuga

**Affiliations:** 1 Endocrinology, Diabetes and Metabolism Division, Department of Internal Medicine, Babcock University Teaching Hospital, Ilishan-Remo, Ogun State, Nigeria; 2 Department of Internal Medicine, Benjamin S. Carson (Snr.) School of Medicine, Babcock University, Ilishan-Remo, Ogun State, Nigeria

## Abstract

**Background:**

Thyrotoxicosis, though commonly encountered in endocrinology practice in Nigeria, seldom presents solely as thyroxine (T4) toxicosis. Thyroid storm, a known life-threatening complication of thyrotoxicosis, can be precipitated by myriad factors. Fine need aspiration for cytology is not known, and has not been previously reported (to the best of our knowledge) to precipitate thyroid storm.

**Case Presentation:**

The case described is that of a 55 year old woman who presented with a neck swelling and features of hyperthyroidism, with biochemical parameters in keeping with T4 toxicosis. Investigating the patient necessitated a fine needle aspiration of the thyroid swelling. The patient thereafter developed hyper-metabolic features, and subsequently progressed to confusion and loss of consciousness few hours following the procedure. Close monitoring and medical management with anti-thyroid and other supportive therapies, resulted in an improved clinical condition.

**Conclusion:**

T4 toxicosis is a distinct biochemical entity of clinical significance. Physicians should bear in mind that micro-trauma from an investigation such as fine needle aspiration of the thyroid gland can tilt a thyrotoxic patient into thyroid storm, and this risk should be considered and prepared for by physicians and patients.

Thyrotoxicosis is a clinical syndrome resulting from elevated free thyroxine (T4) and/or free triiodothyronine (T3) levels, resulting in a generalized acceleration of metabolism. It was initially thought to be a rare finding in blacks (1, 2), with an incidence rate as low as 5.3% seen in Ibadan, Nigeria in a 1972 study (3). However, several recent studies have disproved this assertion, with percentages as high as 36.6% of thyroid disorders in some parts of the country (4), and Graves disease being the commonest etiology(5). Though seldom encountered, the existence of T4 toxicosis has been documented by some clinical researchers (6-9), especially in elderly women (7). T4 toxicosis is characterised by isolated elevation of T4, in the presence of low or normal T3 and low thyroxine stimulating hormone (TSH) in a patient with demonstrable clinical hyperthyroidism (8). Thyroid storm is a life-threatening complication of thyrotoxicosis that can result in mortality as high as 1 in 5-10 cases (10). It is characterised by excessive release of thyroid hormones into circulation, hyperactivity of the adrenergic system, increased sensitivity to catecholamines and increased response to thyroid hormones. It can be precipitated by periods of intense stress such as infection, radioiodine therapy, withdrawal of anti-thyroid drugs, cerebrovascular events, diabetic ketoacidosis, toxemia of pregnancy, trauma, thyroid gland manipulation and emotional stress. Currently, there are a few criteria available, which help to increase the diagnostic accuracy of thyroid storm (11, 12); they will be further elucidated upon in the discussion. 

We report an uncommon variant of thyrotoxicosis, T4 toxicosis, complicated by thyroid storm which was triggered by a previously unrecognized precipitant, fine needle aspiration of the thyroid gland.

## Case Presentation

A 55-year old female Nigerian trader, known hypertensive and type 2 diabetic (diagnosed two years prior to presentation) presented to the emergency room with worsening polyuria and polydipsia, associated with progressive unintentional weight loss, heat intolerance, fatigue and an anterior neck swelling over the past two months. There were no features suggestive of cerebrovascular, cardiac or renal decompensation. Relevant examination findings included a blood pressure of 154/96 mmHg, resting pulse rate of 100 beats per minute, and an anterior neck swelling which was diffuse, firm, not tender, moved with deglutition and not with tongue protrusion. She had a fine distal tremor of outstretched hands. There were no signs of ophthalmopathy, acropachy or dermopathy. Other cardiovascular, respiratory, gastrointestinal and central nervous system (CNS) findings were essentially normal. A random blood glucose of 427mg/dL with glycosuria of 3+ warranted her admission for intravenous (IV) insulin therapy. 

Other routine investigations were essentially normal. However, her glycemic profile remained poorly controlled despite up-titration of insulin and antidiabetic medications (vildagliptin and metformin). An electrocardiogram (ECG) showed sinus tachycardia. Thyroid function test (TFT) showed a markedly elevated free T4 25.9pmol/L (7.2-16.4pmol/L), free T3 was 3.3 pmol/L (3.8-6.0pmol/L) and TSH was 0.330ꙡIU/ml (0.380-5.330). Thyroid ultrasound showed a diffuse thyroid enlargement with multiple colloidal cysts, which necessitated a fine needle aspiration for cytology (FNAC). About 12 hours following the FNAC, the patient started to complain of chills and rigor, as well as profuse sweating, inability to sleep and palpitations. 48 hours later, she became confused, and subsequently drifted into unconsciousness with a Glasgow coma score of 8/15. A diagnosis of thyroid storm was made using the Burch-Wartofsky (score of 85) and Akamizu criteria. She was managed according to 2016 Japan Thyroid Association guidelines for thyroid storm, with the use of carbimazole 20mg 8-hourly, propranolol 40mg 12-hourly, with particular attention paid to supportive care while proceeding with her antidiabetic and antihypertensive drug regimen. Her consciousness subsequently improved. Cytology report showed clusters of benign follicular epithelial cells with round bland nuclei, fine chromatin pattern and moderate cytoplasm on a background containing abundant colloid materials admixed with few leukocytes. The patient made significant clinical improvement in glucose control and thyroid function, and was followed-up on outpatient basis. Post-admission TFT showed reducing free T4 levels, though still elevated (17.3pmol/L) and normal free T3 and TSH (4.8pmol/L; 1.009mIU/L respectively). 

## Discussion

Hyperthyroidism, especially due to Graves’ disease and toxic adenoma are generally more prevalent amongst women globally (13), and a similar pattern is observed in Nigeria (4, 5). T3 toxicosis (a biochemical finding of isolated rise in T3 levels) is an established entity but T4 toxicosis is uncommon. Our report describes a woman with features of hyperthyroidism, an anterior neck mass, elevated T4 with low T3 and TSH. This clinical scenario obviates the metabolic activity of T4, its critical role in maintaining normal body physiology, and the implication of derangements in its serum levels. A recent study has placed the prevalence of T4 toxicosis, in a sample of 548 patients (468 with Graves' disease, 40 had subacute thyroiditis and 40 with toxic adenoma/multinodular goiter), at 6.6%, more than the level quoted for T3 toxicosis (5.6%) (14). Another recent study details a report of a diabetic who presented with features of diabetic ketoacidosis (DKA), T4 toxicosis and thyrotoxic cardiomyopathy occurring at the same time (15). This case is similar to ours in that our patient was also diabetic with a concurrent hyperthyroidism, though our patient did not develop DKA. It is imperative that the managing physician be on the lookout for DKA in a diabetic with hyperthyroidism, as thyrotoxicosis is a known precipitant of DKA and can worsen glycemic control, while DKA is also a known precipitant of thyrotoxicosis and thyroid storm.

One hypothesis as to the pathogenesis of thyroid storm is the increase in the free fraction of thyroid hormones. Most patients from previous studies often show no marked difference in total thyroid hormone levels between patients with thyroid storm and uncomplicated thyrotoxicosis, however the free fractions can be higher in thyroid storm (16), a finding similar to what was observed in our report. TSH has been found to be the most sensitive, and perhaps the only test needed to determine the biochemical function of the thyroid gland (17). In our study, TSH was consistently low in the TFTs, confirming a primary hyperthyroidism. This is further buttressed by the thyroid ultrasound findings of a colloid goiter in our patient. The use of radioactive iodine for diagnostic and therapeutic purposes, as well as the biochemical estimation of iodine levels were not done in our patient because of the unavailability of these highly specialized methods. These however did not preclude the appropriate clinical management of the case, as other available diagnostic and treatment modalities were put to use, culminating in good recovery of the patient.

The two commonly referenced tools in aiding diagnosis of thyroid storm are the Burch-Wartofsky scale and the Akamizu criteria (11, 12), which were combined by the authors of this report in making a diagnosis of thyroid storm. The authors chose to combine both tools, as not much work has been done to repeatedly validate them. This strategy will tend to increase the chances of making a correct diagnosis of thyroid storm.

Thyroid storm has been well documented to have resulted from trauma from vehicular accidents (18), manipulation/vigorous palpation of the gland (19), strangulation (20), and even direct blunt trauma to the gland (21). A case of transient hyperthyroidism was recently reported following thyroid ultrasound (22). However thyroid storm complicating FNAC of the thyroid gland has not been previously documented to the best of our knowledge. The reasons for this may include - the procedure not being thought by many to be significantly traumatic, and attribution of thyroid storm to other precipitants or misses due to failure to recognize a patient as having thyroid storm following the procedure. The technique for fine needle aspiration of the thyroid gland involves inserting a 22-27 gauge needle into the thyroid to obtain cells (or fluid) for cytology. 

The diagnosis of thyroid storm is clinical, as there is no cut-off of serum thyroid hormones that differentiates a severe thyrotoxicosis from thyroid storm. The clinician should not be preoccupied with differentiating a severe thyrotoxicosis from thyroid storm using the various criteria available, but should approach the care of the patient in an aggressive manner to reduce morbidity and prevent mortality, which can be as much as 20% in some cases (23). The guidelines for management of thyroid storm by the Japan Thyroid Association and Japan Endocrine Society was adopted in the management of our patient (24). The guideline advocates supportive care to mitigate the effects of systemic decompensation, antithyroid drugs targeted at reducing the biosynthesis of thyroid hormones, halting the release of stored thyroid hormone from the gland, preventing peripheral conversion of T4 to T3, and anti-adrenergic medications aimed at controlling the adrenergic symptoms associated with thyrotoxicosis. Antibiotics and corticosteroids are also useful adjunctive therapies.

Thyroliberin (thyroid releasing hormone) stimulation test, radioactive iodine test and thyroid antibodies were not done due to the unavailability of these tests in the environment where this case was managed, and financial constraints. However the diagnosis of thyrotoxicosis was not in doubt in spite of this, as the patient had obvious clinical features in keeping with hyperthyroidism, as well as a supportive biochemical picture.

This case report draws attention to the existence of T4 toxicosis as a distinct biochemical variant of hyperthyroidism, with clinical implications. It also highlights the risk of thyroid storm developing from an investigative procedure in a patient with a toxic thyroid mass. It is recommended that the patient and physician be aware of this risk, and be adequately prepared to mitigate any possible adverse manifestation.
